# Differential expression of proteins in genetically distinct *Trypanosoma cruzi* samples (TcI and TcII DTUs) isolated from chronic Chagas disease cardiac patients

**DOI:** 10.1186/s13071-018-3181-1

**Published:** 2018-11-29

**Authors:** Maykon Tavares de Oliveira, Karina Taciana Santos Silva, Leandro Xavier Neves, Max Jean de Ornelas Toledo, William Castro-Borges, Marta de Lana

**Affiliations:** 1 Programa de Pós-Graduação em Ciências Biológicas do Núcleo de Pesquisas em Ciências Biológicas (NUPEB), Campus Universitário Morro do Cruzeiro, Universidade Federal de Ouro Preto, CEP, Ouro Preto, MG 35400-000 Brazil; 2 Departamento de Farmácia, Escola de Farmácia, Campus Universitário Morro do Cruzeiro, Universidade Federal de Ouro Preto, CEP, Ouro Preto, MG 35400-000 Brazil; 3 Programa de Pós-Graduação em Biotecnologia do Núcleo de Pesquisas em Ciências Biológicas (NUPEB), Campus Universitário Morro do Cruzeiro, Universidade Federal de Ouro Preto, CEP: 35400-000, Ouro Preto, MG Brazil; 4 Departamento de Ciências Básicas da Saúde – Parasitologia, Universidade Estadual de Maringá, CEP: 87020-900, Maringá, Paraná, PR Brazil; 5 Departamento de Ciências Biológicas, Instituto de Ciências Exatas e Biológicas, Campus Universitário Morro do Cruzeiro, Universidade Federal de Ouro Preto, CEP: 35400-000, Ouro Preto, MG Brazil; 6 Departamento de Análises Clínicas, Escola de Farmácia, Campus Universitário Morro do Cruzeiro, Universidade Federal de Ouro Preto, CEP: 35400-000, Ouro Preto, MG Brazil

**Keywords:** *Trypanosoma cruzi*, DTUs, Differential proteomics, Chagas disease

## Abstract

**Background:**

*Trypanosoma cruzi*, a hemoflagellate protozoan parasite and the etiological agent of Chagas disease (CD), exhibits great genetic and biological diversity. Infected individuals may present clinical manifestations with different levels of severity. Several hypotheses have been proposed to attempt to correlate the diversity of clinical signs and symptoms to the genetic variability of *T. cruzi*. This work aimed to investigate the differential expression of proteins from two distinct genetic groups of *T. cruzi* (discrete typing units TcI and TcII), isolated from chronically infected individuals displaying the cardiac form of CD. For this purpose, epimastigote forms of the two isolates were cultured *in vitro* and the cells recovered for protein extraction. Comparative two-dimensional (2D) gel electrophoreses were performed and differentially expressed spots selected for identification by mass spectrometry, followed by database searching and protein categorization.

**Results:**

The 2D electrophoretic profiles revealed the complex composition of the *T. cruzi* extracted proteome. Protein spots were distributed along the entire pH and molecular mass ranges attesting for the integrity of the protein preparations. In total, 46 differentially expressed proteins were identified present in 40 distinct spots found in the comparative gel analyses. Of these, 16 displayed upregulation in the gel from TcI-typed parasites and 24 appeared overexpressed in the gel from TcII-typed parasites. Functional characterization of differentially expressed proteins revealed major alterations associated with stress response, lipid and amino acid metabolism in parasites of the TcII isolate, whilst those proteins upregulated in the TcI sample were primarily linked to central metabolic pathways.

**Conclusions:**

The comparative 2D-gel electrophoresis allowed detection of major differences in protein expression between two *T. cruzi* isolates, belonging to the TcI and TcII genotypes. Our findings suggest that patients displaying the cardiac form of the disease harbor parasites capable of exhibiting distinct proteomic profiles. This should be of relevance to disease prognosis and treatment.

**Electronic supplementary material:**

The online version of this article (10.1186/s13071-018-3181-1) contains supplementary material, which is available to authorized users.

## Background

Chagas disease is caused by the hemoflagellate protozoan parasite *Trypanosoma cruzi*. According to data from the World Health Organization [1], 6–7 million individuals are infected by the parasite throughout Latin America and around 60–70 million people are at risk of infection. Hampered by the high genetic variability of the parasite, the afflicted population exhibits varying clinical forms of the disease [2–4]. These are mostly refractory to treatment, particularly for chronically infected individuals [5].

It has been established that the natural populations of *T. cruzi* are represented by at least seven discrete typing units (DTUs) TcI-TcVI and Tcbat [6]. Any possible correlation between these DTUs and the occurrence of the different clinical forms observed in chagasic patients is, to date, quite controversial [7, 8]. Since regulation of gene expression in trypanosomatids operates post-transcriptionally, molecular approaches to analyze and compare the proteomes of *T. cruzi* populations may be fundamental to show the association between their genetic variability and the clinical manifestations of Chagas disease [9].

In this regard, Telleria et al. [5] pioneered the use of 2D-gel electrophoresis to highlight differential protein expression among nine *T. cruzi* laboratory-cloned stocks. Differences in spot volumes for several proteins allowed the authors to highlight proteomic variability among the clones and their grouping into four distinct DTUs. However, only a few representative spots (nine in total) of major soluble components such as α-/β-tubulins and heat-shock proteins were confidently identified by mass spectrometry. This prevented major conclusions to be drawn concerning the biological meaning of the proteomic dissimilarities observed for the distinct DTUs. A more recent approach revealed that mass spectral libraries generated with *T. cruzi* stocks representatives of the distinct DTUs can be used to confidently distinguish among the genotypes [10].

Towards a patient-oriented approach, we have previously reported a range of biological properties associated to *T. cruzi* samples that were isolated from chronically-infected individuals displaying the cardiac, indeterminate and digestive forms of Chagas disease. Briefly, blood samples were collected from patients living in Minas Gerais, Brazil, and the recovered parasites genotyped as belonging to DTUs I, II and VI. Marked differences in their biological behaviour were observed when samples belonging to these three DTUs were grown *in vitro* or used to infect Vero cells [11]. Moreover, *in vivo* studies of parasitemia, polymorphism of trypomastigotes, cardiac inflammation and fibrosis contributed to pinpoint major differences among those DTUs in the murine model of infection.

In the present study, aiming to provide molecular insights into the dissimilarities observed, we have selected two of those strains belonging to the distinct and phylogenetically polar groups (TcI and TcII) for a comparative 2D-gel electrophoretic analysis. Mass spectrometry identification greatly expanded the number and the cellular processes associated with differentially expressed proteins observed between these two genetic groups. Understanding the role such proteins play during parasitism should be of relevance to disease outcome and treatment, in particular for cardiac chagasic patients.

## Methods

### Selection of Chagas disease patients

The two *T. cruzi* samples used in this study were isolated from chronic Chagas disease patients (patients code: PR150 and 452), living in different regions in the state of Minas Gerais (MG), Brazil. The PR150 sample (TcI) was isolated from a female patient, living in Januária, northern MG [12], and sample 452 (TcII) was isolated from a patient living in Berilo, Vale do Jequitinhonha, MG [13]. These two locations are geographically separated by 487 km. Both patients presented the cardiac clinical form of the disease (moderate and severe, respectively) and agreed to participate in the Chagas Disease Programme.

The serological diagnosis for *T. cruzi* infection was confirmed according to the guidelines of the Ministry of Health of Brazil [14] and the WHO [15]. In addition, the patients were evaluated through anamnesis, clinical examination, posteroanterior chest X-ray, contrast-enhanced X-ray of the esophagus and colon, electrocardiogram and echocardiogram. After analyzing the results, the patients were clinically classified according to the Brazilian Consensus on Chagas Disease of 2015 [16].

### Obtaining *T. cruzi* isolates

*Trypanosoma cruzi* isolates were obtained by using the blood culture technique described by Chiari et al. [17]. Briefly, sterile tubes containing heparin (Vacuntainer BD, New Jersey, USA) were used to collect approximately 30 ml of intravenous blood. The blood was transferred to a conical tube and immediately centrifuged at 2070*× g* for 10 min at room temperature. To the red blood cell (RBC) concentrate obtained, 5 ml of LIT medium [18] was added and followed by a new centrifugation step performed under the same conditions. This procedure was then repeated to obtain a clean leukocyte layer that was removed for culture. The RBC layer was resuspended in 5 ml of LIT medium and subdivided into three new tubes containing 5 ml LIT medium each. These tubes were incubated at 28 °C and homogenized manually three times a week. One drop of the pellet from each preparation was analyzed under an optical microscope at 30, 60, 90 and 120 days after collection to detect the presence of multiplying parasites.

### Acellular culture of *T. cruzi*

*Trypanosoma cruzi* isolates were maintained in exponential growth by successive addition of LIT medium until approximately 35 ml of culture were obtained. The culture was transferred to tapered tubes and centrifuged at 2070× *g* at 4 °C for 30 min. After centrifugation, the supernatant was carefully discarded by inversion and 10 ml of sterile buffered saline (PBS, pH 7.2) was added to the pellet. The mixture was subjected to a new centrifugation step as before for 15 min. After repeating this procedure, the pellet was transferred to a pre-weighed 1.5 ml microcentrifuge tube. The pellet was then washed with sterile PBS and centrifuged at 6900*× g* for 10 min. The supernatant was carefully removed, the tube re-weighed, and the wet mass of parasites stored at -70 °C for further proteomic analysis. This protocol was repeated for each of the three biological replicates used in this study.

### Protein extraction

Approximately 100 mg of epimastigote cell pellets were used for the extraction of proteins. The samples were homogenized in 500 μl rehydration solution (7 M urea, 2 M thiourea, 2% w/v CHAPS, 0.002% w/v bromophenol blue; all from Sigma-Aldrich, Missouri, USA) containing 1× Protease Inhibitor Cocktail (Sigma-Aldrich) at a final dilution of 1:25, for 10 min in an ice bath. The samples were then sonicated by means of a series of three 15 s pulses, each followed by 45 s resting on ice between cycles. Homogenates were centrifuged at 20,000× *g* for 1.5 h at 4 °C to remove cell debris. The 1D electrophoretic profiles were used to verify the integrity of the extracts and to normalize, through densitometric analysis (software Quantity one v.29.0), the total amount of protein present in each sample. Briefly, a 5 μl aliquot of each sample was separated under denaturing conditions using 12% SDS-PAGE. After 30 min in fixative solution (40% ethanol/7% acetic acid), the gel was stained in 0.02% Coomassie Blue G-250 solution (Sigma-Aldrich) for 2 h.

### Two-dimensional gel electrophoresis

2D-gels were prepared using 150 μg of proteins obtained for each isolate. First, a precipitation step was performed on trichloroacetic acid/acetone (1:8), followed by solubilization of proteins in rehydration buffer containing 1% DTT and 0.8% ampholytes (pH3-10 NL Buffer IPG; GE Healthcare, Uppsala, Sweden) at a final volume of 250 μl for each sample. The first dimension was performed using 13 cm gel strips (pH3-10 NL, GE Healthcare). Proteins were isoelectrofocalized at 23 °C using IPGphor 3 (GE Healthcare) according to the following protocol: step 1, 14 h of passive rehydration; step 2, 1 h at constant 500 V; step 3, gradient up to 1000 V in 1 h; step 4, gradient up to 8000 V for 2.5 h; step 5, held at constant 8000 V until making a total of 29,500 V throughout the isoelectrofocalization protocol. All steps were performed at a maximum current of 50 μA per strip. The isoelectrofocalized proteins were then reduced, followed by alkylation using 1% DTT and 4% iodoacetamide, respectively, in equilibration buffer (6 M urea, 75 mM Tris-HCl pH 8.8, 29.3% glycerol, 2% SDS, 0.002% bromophenol blue) as described previously [19]. The second dimension was performed at 5 °C on 12% SDS-PAGE gel (18 × 16 cm) at 20 mA/gel, for approximately 6 h. The gels were fixed in 2% v/v orthophosphoric acid/30% v/v ethanol overnight, and then washed 3 × 10 min with 2% v/v orthophosphoric acid. Staining was performed for 48 h in a Coomassie colloidal solution containing 2% v/v orthophosphoric acid, 18% v/v ethanol, 15% w/v ammonium sulfate and 0.002% w/v Coomassie blue G-250 (Sigma-Aldrich). Excess stain was removed by washing the gels in 20% v/v ethanol for 5 min before they were scanned using ImageScanner III (GE Healthcare).

Densitometric analyses of 2D-gels were performed using the SameSpots software (TotalLab Ltd., v.20, UK) following the manufacturer's recommendations. Quantitative analyses were performed on spots that presented ≥ 1.5-fold change in comparison to the respective spots found in the gel used as reference (the one from strain 452). The maximum standard deviation allowed per spot, for triplicate gels, was 10%. The spots that met these criteria were selected for protein identification by mass spectrometry.

### Protein identification by mass spectrometry and database searching

Selected spots were manually excised and destained in 40% v/v ethanol/7% v/v acetic acid. After washing in ultrapure water they were submitted to gel digestion according to Helman et al. [20]. The recovered peptides were analyzed on a Q-Exactive hybrid quadrupole-orbitrap mass spectrometer (Thermo Fisher Scientific, Bremen, Germany). Seventeen μl of peptide samples were injected into a nanoUHPLC instrument (Dionex UltiMate 3000, Thermo Fisher Scientific) through a trapping system (Acclaim PepMap100, 100 μm × 2 cm, C18, 5 μm, 100 A, Thermo Scientific) for 3 min using as solvent 98% water/2% acetonitrile (ACN) with 0.05% trifluoracetic acid (TFA) and subsequently directed into a capillary column (Acclaim PepMap100, 75 μm × 25 cm, C18, 3 μm, 100 A, Thermo Fisher Scientific). Reversed-phase separation of peptides was performed at 40 °C in a gradient of solvent A (water, 0.1% formic acid) and B (80% ACN / 20% water, 0.1% formic acid), at a flow rate of 0.3 μl/min. Peptides were sequentially eluted over a gradient spanning from 4% to 15% of solvent B for 2 min, increasing to 55% of B over additional 15 min. Then, peptides were directed to the online coupled mass spectrometer by means of a nanospray ionization source. Ions were detected under positive mode through data dependent analysis. Resolution for precursor ions (MS1) was set to 70,000 (FWHM at 200 *m*/*z*) with an automatic gain control target of 3e^6^, maximum injection time of 100 ms, scanning over 300–2000 *m/z*. The Top10 most intense precursor ions of each MS1 mass spectra were individually isolated with a 2.0 Th window for activation *via* higher-energy collisional dissociation (HCD) with normalized energy of 30 V. Only peptides exhibiting charge states of +2, +3, +4 and +5 were selected. Automatic gain control target was set to 5e^5^ (minimum accumulation of 3.3e^3^) with maximum injection time of 75 ms. Dynamic exclusion of 15 s was active.

MS1 and MS2 spectral data were submitted to Proteome Discoverer v.2.1 (Thermo Fischer Scientific) platform, underpinned by the SEQUEST HT searching algorithm, using a compilation of *T. cruzi* databases downloaded from TriTrypDB (available at http://tritrypdb.org/tritrypdb/ and containing 21,060 sequences/10,322,012 residues). This combined database contained equivalent number of sequences from TcI- and TcII-typed parasites. Search parameters included cysteine carbamidomethylation as a fixed modification, methionine oxidation as a variable modification, up to one trypsin missed cleavage site, tolerance set to ± 10 ppm for precursor and ± 0.1 Da for product ions. Only proteins identified with peptides exhibiting expected values ≤ 0.05, false discovery rate ≤ 1% and percentage coverage ≥ 8%, with at least one unique peptide, were considered in this study. Whenever more than one protein identification were assigned to a single spot, the major component was associated with the higher total Area Under Curve (AUC) observed, and only if the total AUC of a second protein represented at least 40% of the major area, it was indicated.

### Functional categorization of proteins

The identified proteins were distributed into 9 categories, according to their biological function and termed as follows: transcription and translation, heat-shock response, protein synthesis and degradation, metabolism (related to carbohydrates, nucleotides, amino acids and lipids), cytoskeleton and cell signaling. Gene Ontology information (available at http://geneontology.org/) was used to support the protein categorization.

### Statistical analysis

Data derived from the quantitative proteomic analyses were examined using the Graph Pad Prism software (v.5). The comparison between the two distinct groups of gels was made through ANOVA. The significance level adopted was *P* ≤ 0.05.

## Results

### Proteomic analysis of epimastigote forms of *T. cruzi*

**Fig. 1:**
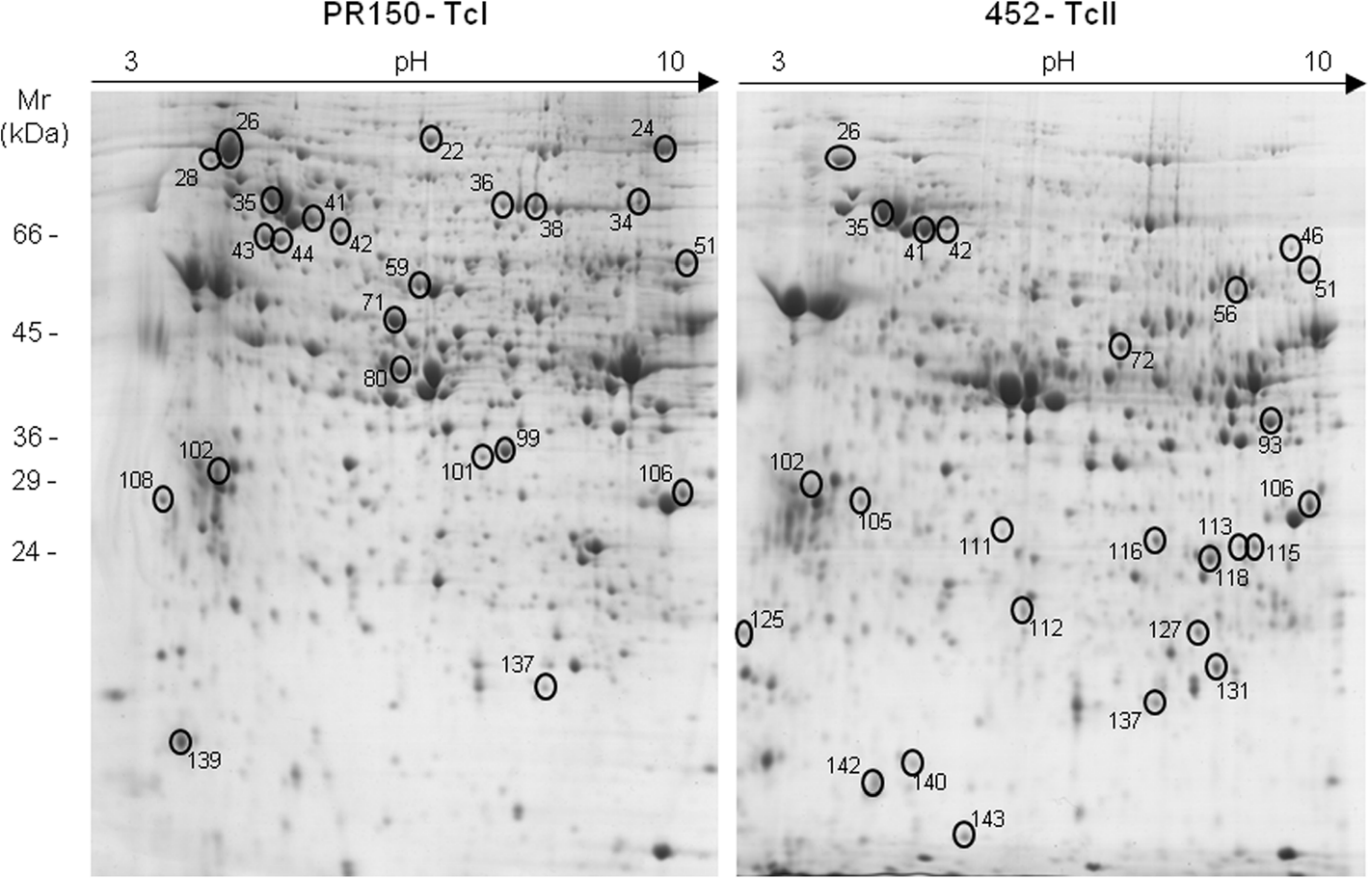
Comparative two-dimensional gel electrophoretic profiles for samples TcI (PR150) and TcII (452). Protein fractions from both samples were isoelectrofocalized on a 13 cm pH3-10NL IPG strip and subsequently proteins separated according to their molecular masses using 12% SDS-PAGE. After Coomassie staining, the gel images were aligned using the SameSpots software for identification of differentially expressed spots (numbered). These were then processed for in gel digestion and the resulting peptides submitted to mass spectrometric identification. The figure is a representative of a pair of gels

The protein extracts obtained from three distinct epimastigote samples resulting from the acellular cultivation of each strain (PR150-TcI and 452-TcII) were submitted to isoelectrofocalization and subsequent bidimensional electrophoresis in 13 cm gel, pH3-10NL. Following Coomassie staining, the gels obtained in triplicates were analyzed using the SameSpots software. Overall, good reproducibility for the gels was achieved, with proteins covering the entire pH and molecular mass ranges indicating adequate protein integrity following extraction (Fig. 1).

Over 1700 spots were detected, with approximately 200 displaying ≥ 1.5 fold change for each overlapping gel pair. The comparison among the triplicates revealed 40 matched spots showing < 10% standard deviation. Among them, 16 protein spots from gel PR150 appeared upregulated relative to their corresponding spots in the gel from strain 452, whereas 24 were found upregulated in gel 452 relative to their respective pairs in gel PR150.

The spots revealing expression differences between strains PR150 and 452 were then excised, digested with trypsin and subjected to protein identification by mass spectrometry. Positive identifications were categorized according to their molecular function as shown in Table 1 (see also Additional file 1: Table S1 for mass spectrometric data related to peptide/protein identifications).

**Table 1:** Identities associated with differentially expressed proteins in the comparison between TcI and TcII genotypes

Spot no./Strain	Accession number	Description	Gene	Unique peptides	Coverage (%)	pI	Observed MW (kDa)
Translation/Transcription							
22/PR150	TCSYLVIO_990036	Elongation factor 2	*EF2*	31	42.25	6.04	75.70
101/PR150	TcCLB.503419.50	RNA-binding protein RGGm	*RGG2*	8	27.51	8.76	34.45
139/PR150	TcCLB.506925.130	Eukaryotic translation initiation factor 5a	*EIF5A*	7	58.68	5.00	9.88
56/452	TcCLB.509583.10	Chaperonin TCP20	*TP20*	22	41.07	7.69	51.11
72/452	TCSYLVIO_002198	Elongation factor 1-alpha (EF-1-alpha)	*EF1A*	1	44.79	8.98	43.99
Heat-shock response							
28/PR150	TCSYLVIO_008621	Heat shock protein 85	*HSP85*	6	10.99	6.35	73.76
43/PR150	TCSYLVIO_004969	Chaperonin HSP60, mitochondrial precursor	*HSP60*	44	66.55	5.50	65.35
44/PR150	TCSYLVIO_004969	Chaperonin HSP60, mithocondrial precursor	*HSP60*	36	75.09	5.50	65.35
71/PR150	TCSYLVIO_004831	Thiol-dependent reductase 1	*TDR1*	11	36.63	5.97	46.04
26/452	TcCLB.507713.30	Heat-shock protein 85	*HSP85*	33	43.18	5.15	74.41
35/452	TcCLB.511211.170	Heat-shock protein 70 (HSP70)	*HSP70*	6	48.01	5.57	69.23
41/452	TCSYLVIO_003281	Heat-shock 70 kDa protein, mithocondrial precursor	*HSP70*	33	46.56	6.13	67.29
42/452	TCSYLVIO_003281	Heat-shock 70 kDa protein, mithocondrial precursor	*HSP70*	15	27.94	6.13	66.00
125/452	TcCLB.506207.50	IgE-dependent histamine-releasing factor	*HRF*	4	16.47	4.64	18.35
131/452	TcCLB.506207.50	IgE-dependent histamine-releasing factor	*HRF*	6	21.18	4.64	16.00
131/452	TcCLB.509775.40	Iron superoxide dismutase	*SODB*	3	56.65	8.60	16.00
140/452	TcCLB.503.899.130	Glutathione peroxidase-like protein	*GPX*	8	39.33	6.00	8.47
Cell signalling							
105/452	TcCLB.503855.20	Spermidine synthase	*SPSYN*	1	35.14	5.41	27.61
140/452	TcCLB.503715.30	Ras-related protein RAB1A	*RAB1A*	3	15.71	7.77	8.47
143/452	TcCLB.511407.60	Small GTP-binding protein RAB11	*RAB11*	7	43.78	8.13	3.36
Protein synthesis/Degradation							
59/PR150	TCSYLVIO_005562	Hypothetical protein (Metalocarboxypeptidase1)	*MCP1*	19	50.21	5.86	48.66
108/PR150	TCSYLVIO_001046	Peptidase M20/M25/M40	*MT2598*	8	17.78	5.43	27.61
137/PR150	TcCLB.507639.40	Proteasome beta 5 subunit	*PSMB5*	9	27.01	5.90	13.47
127/452	TCSYLVIO_005774	Proteasome beta 2 subunit	*PSMB2*	9	24.66	9.19	18.35
Carbohydrate metabolism							
24/PR150	TCSYLVIO_008551	Pyruvate phosphate dikinase	*PPDK*	3	37.18	6.73	75.05
99/PR150	TCSYLVIO_002700	Cytosolic malate dehydrogenase	*MDH*	9	35.24	6.73	34.45
42/452	TCSYLVIO_004608	Phosphoglycerate mutase	*PGMI*	26	61.01	5.92	66.00
51/452	TcCLB.507547.90	Glycosomal phosphoenolpyruvate carboxykinase	*PEPCK*	18	38.86	8.37	62.76
106/452	TcCLB.510105.230	Glyceraldehyde 3-phosphate dehydrogenase	*GAPDH*	19	55.92	8.46	28.07
Nucleotide metabolism							
34/PR150	TCSYLVIO_003291	Tetrahydrofolate synthase	*MTHFD1*	10	16.67	7.68	69.23
46/452	TcCLB.508731.60	Adenylosuccinate synthetase	*ADSS*	7	45.13	8.21	63.46
Aminoacid metabolism							
34/PR150	TCSYLVIO_006380	Urocanate hydratase	*UROC1*	10	14.67	6.86	69.23
36/PR150	TCSYLVIO_006380	Urocanate hydratase	*UROC1*	16	20.74	6.86	69.23
38/PR150	TCSYLVIO_006380	Urocanate hydratase	*UROC1*	37	44.74	6.86	68.58
80/PR150	TCSYLVIO_000769	Tyrosine aminotransferase	*TAT*	7	49.05	6.54	42.38
56/452	TCSYLVIO_004599	Histidine ammonia-lyase	*HAL*	16	29.59	8.34	51.11
93/452	TCSYLVIO_001538	Aspartate aminotransferase	*GOT1*	13	42.34	8.81	36.50
111/452	TCSYLVIO_006042	Arginase	*ARG*	12	54.22	6.46	25.88
112/452	TCSYLVIO_003182	Pyridoxal kinase	*PDXK*	1	23.00	6.44	20.23
Lipid metabolism							
113/452	TcCLB.506213.50	Prostaglandin F synthase	*PGFS*	4	15.66	8.82	24.00
115/452	TcCLB.511693.90	Electron-transfer-flavoprotein, alpha polypeptide	*ETFA*	4	57.01	8.27	24.00
116/452	TcCLB.506213.50	Prostaglandin F synthase	*PGFS*	8	30.25	8.82	24.00
118/452	TcCLB.510105.240	Short chain 3-hydroxyacyl-coa dehydrogenase	*HCD2*	9	34.69	8.63	23.50
Cytoskeleton							
28/PR150	TCSYLVIO_007343	Calpain-like cysteine peptidase	*SMP-1*	10	15.03	5.05	73.76
102/452	TcCLB.506563.40	Beta tubulin	*TUBB*	9	33.26	4.81	29.92
142/452	TCSYLVIO_008641	Epsilon tubulin	*TUBE1*	6	15.58	6.52	7.05

**Fig. 2:**
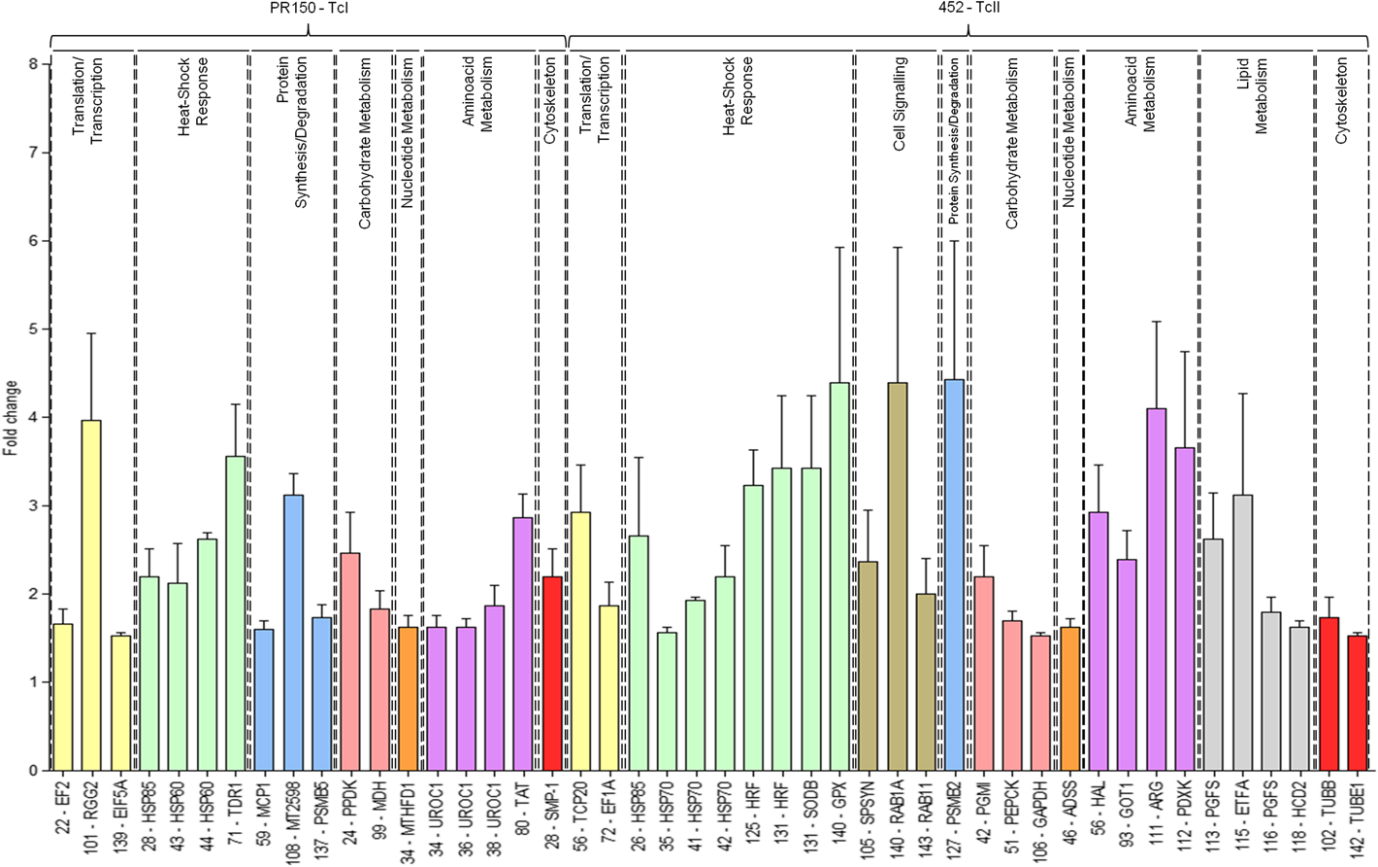
Fold changes associated with differentially expressed proteins in the comparison between TcI (PR150) and TcII (452) genotypes. Spot volumes associated with the differentially expressed proteins were processed by the SameSpots software and the ratio obtained between them, for corresponding spots in each gel, provided its respective fold change. The gel spots from sample 452 were taken as reference. Only positive fold values are shown and protein identities associated with each genotype are grouped (in curly brackets)

Fold changes in expression for each protein described in Table 1 were represented in a bar plot. As shown in Fig. 2, proteins belonging to diverse functional groups had their levels of expression altered when compared between the two evaluated genotypes. Notably, the representative sample of the TcII genotype (patient 452 exhibiting severe cardiac clinical form) demonstrated differential expression of proteins related to stress response and metabolism of amino acids and lipids. Concerning the representative TcI genotype (patient PR150 exhibiting moderate cardiac clinical form), there was pronounced expression of proteins linked to central metabolic pathways.

## Discussion

Changes in expression of proteins related to stress response are genetically correlated with biological aspects of *T. cruzi* infection. Among the identified proteins, three presented increased expression in the PR150 (TcI) sample [heat-shock protein 85 (Hsp85), heat-shock protein 60 – (Hsp60) and thiol-dependent reductase 1 (TDR1)], and five proteins, known to perform related molecular functions, were also found at higher levels in strain 452 (TcII) [heat-shock proteins 85 and 70 (Hsp85 and Hsp70, respectively), IgE-dependent histamine-releasing factor (HRF), iron superoxide dismutase (SODB) and glutathione peroxidase-like protein (GPX)].

As for heat-shock proteins, Urményi et al. [21] reviewed their relevance in the context of *T. cruzi* biology. It is known that this protozoan parasite has a complex life-cycle involving invertebrate and vertebrate hosts being subject to several types of stress. Thus, molecular chaperones and proteases are highly expressed in order to assist in the maintenance of cellular homeostasis. Some families of HSPs, especially Hsp70, Hsp90, Hsp100, Hsp40 and small HSPs, have conserved and unique sequences for *T. cruzi* and demonstrate specific expression patterns among the different strains. In addition, the different expression patterns observed are relevant to parasite biology, particularly concerning resistance to treatment [21, 22]. It has also been demonstrated that parasites expressing higher levels of HSPs have a greater capacity to transit between the wild and domestic cycle of Chagas disease, since they present greater multiplication and metacyclogenesis in the triatomine vector. This favors an enhanced capacity for infection of the vertebrate hosts (man and domestic/wild animals) through the mechanism of natural transmission. This family of proteins has been associated with mechanisms of intracellular survival, metacyclogenesis and virulence [21, 23–25].

According to previous work by our research group, PR150 (TcI) and 452 (TcII) strains have demonstrated capacity to infect Vero cells [11]. However, when we compared the infection in the murine model, only strain 452 (TcII) was able to infect mice and generate patent parasitemia. This might be related to a higher expression of heat-shock proteins and other molecules related to stress resistance shown herein. Several studies have reported that TcII parasites isolated from humans are, in fact, the most likely to infect and present patent parasitemia in mice [26, 27]. Therefore, they are more capable of infecting vectors in view of this characteristic and the predominance of larger blood trypomastigotes in acute, subacute and chronic infections successively. This, in turn, facilitates the infectivity to new vectors, again allowing parasite dissemination in nature [28]. In addition to protecting the parasite against thermal stress, such proteins play an important role during the oxidative burst that occurs during infection of mammalian cells by *T. cruzi* [21, 25]. During this process, the parasite is phagocytosed by macrophages and retained in parasitic vacuoles where they are attacked by lysosomal enzymes and reactive oxygen species. Thus, strain 452 (TcII) parasites can escape from the parasitic vacuole with greater ease, ensuring their intracellular life-cycle [21] by multiplying more intensely in the host and, consequently, resulting in higher levels of parasitemia [8, 26, 27].

TDR1, also classified as a stress response protein and identified in the PR150 (TcI) strain, is homologous to TC52 protein in *T. cruzi*. This protein is also related to parasite’s protection against oxidative stress during host cell infection [29]. A study that evaluated TDR1 expression in different strains of *T. cruzi* submitted to treatment with benznidazole observed higher expression in strains considered resistant to the specific treatment, typical of TcI samples [30]. The same authors also suggested that strains or clones that overexpress TDR1 can escape from the immune system more easily and have high virulence [7, 31].

Another important overexpressed protein in strain 452 (TcII) is IgE-dependent histamine-releasing factor. This protein has been linked to the ability of parasites to generate chronic inflammation [32]. This increase in expression may explain the pronounced ability of this strain to generate inflammation in the cardiac tissue of infected mice, both in the acute and chronic phases of infection [10]. In humans, TcII parasites cause more marked pathology due to inflammation later replaced by fibrosis, mainly in the heart [33, 34]. In addition, the expression of this gene has been associated with chronic inflammatory processes of the airways, such as allergic responses and asthmatic symptoms [35].

The enzymes SODB and GPX, both overexpressed in the TcII strain (452), confer protection against oxidative stress. Their expression is frequently related to an amplified immune response and consequent generation of reactive oxygen species, justifying their relevance in the pathogenesis of Chagas disease [36, 37].

The SOD enzymes catalyze the conversion of superoxide radicals (O_2_^-.^) into oxygen (O_2_) and hydrogen peroxide (H_2_O_2_). Thus, for *T. cruzi* parasites they provide the ability to withstand the highly oxidative environment rich in NADPH oxidase and nitric oxide synthase found in macrophages during phagocytosis [38]. It was demonstrated by Martinez et al. [39] and Piacenza et al. [40] that expression of SODB isoform correlates with high levels of resistance to oxidant treatment when compared to other isoforms (SODA and SODC) found in *T. cruzi*.

In terms of central metabolic pathways we observed increased expression of proteins linked to the metabolism of amino acids [histidine ammonia lyase (HAL), aspartate aminotransferase (GOT1), arginase (ARG) and pyridoxal kinase PDXK)] and lipids [prostaglandin F-synthase (PGFS), electron transfer flavoprotein (ETFA) and 3-hydroxyacyl-Coa dehydrogenase (HCD2)] in the TcII strain (452) relative to the TcI strain (PR150).

Trypanosomatids alternate the main form of energy production according to the availability of substrates found in the environment. When sugar is available, *T. cruzi* uses aerobic fermentation to metabolize it. However, whenever needed, it can activate the mitochondrial pathway and oxidize amino acids with the concomitant production of ammonia for energy generation [41–43]. It is also postulated that amino acid metabolism is involved with the cytosolic reoxidation of NADH and the recycling of methionine [44]. The amino acid proline appears to be very closely associated with *T. cruzi* metacyclogenesis when the parasite is present on a nutrient-poor environment (e.g. absence of blood supply in the vector) or an aged culture medium [22, 45].

Glutamate is also another important amino acid used in oxidative metabolism and histidine is one of its precursors [41]. The biochemical pathway involved in such conversion employs some of the enzymes identified in this work as HAL and UROC1; these are commonly found overexpressed in the *T. cruzi* stages involving the insect vector [46]. Proline, another amino acid metabolically related to glutamate, seems to contribute through the supply of energy, allowing parasite growth in energetically unfavorable environments [47]. Additionally, proline has been linked to resistance to oxidative stress [48]. Aspartate aminotransferases carry out amino acid transamination producing 2-oxo acids used up in the Citric acid cycle. In addition, they apparently have the ability to convert 2-oxo-4-methylbutyrate to methionine, thus participating in the protection against oxidative stress [49, 50].

Arginase is one of the enzymes responsible for competing with iNOS to counterbalance the defensive action of NO in infectious diseases by promoting parasite proliferation and differentiation. In addition, the enzyme suppresses T cell response through the production of polyamines with anti-inflammatory and immunosuppressive activities and influences, through arginine metabolism, the relationship between innate and acquired immune responses [51–53]. Although arginase activity has proven essential for *T. cruzi* infection, our study provides relevant data on the differential expression of this enzyme associated to a specific genotype. This observation merits attention and could have implications for the proposal of novel therapeutic approaches (e.g. arginase inhibitors) for patients harboring parasites belonging to distinct DTUs.

Overexpression of the PDXK enzyme associated with the TcII strain is also of interest. It is involved in the production of vitamin B6 (pyridoxal 5'-phosphate) from its precursors pyridoxal, pyridoxamine and pyridoxine [54]. This vitamin is a coenzyme for transaminases, assuming a crucial role in transamination, decarboxylation, racemization and amino acid substitution reactions, as well as being implicated in the antioxidant defense [55].

As for lipid metabolism, ETFA and HCD2 proteins are involved in the generation of ATP through β-oxidation of fatty acids. In its turn, PGFS oxidoreductase plays an important role in evasion of the host immune response through prostaglandin synthesis, as demonstrated in *Leishmania infantum* by Araújo-Santos et al. [56]. The ability of *T. cruzi* to metabolize arachidonic acid into eicosanoids has been demonstrated to play an important role in parasite invasion and survival in the host [57–60].

Our comparative 2D-gel electrophoresis provided a reliable tool to visualize and measure pronounced differences in protein expression associated to distinct *T. cruzi* genotypes (TcI and TcII), isolated from chronically infected patients displaying the cardiac form of CD. The high levels of proteins associated to stress response and the key aspects of amino acid and lipid pathways overrepresented in the TcII genotype might contribute to explain the differential biological behavior observed for strain 452 during our preliminary *in vitro* and *in vivo* studies [10].

## Conclusions

Here we have expanded the repertoire of protein molecules distinctly expressed in the two *T. cruzi* genotypes mostly associated to disease in South America, particularly in the Brazilian population [59]. The search for effective methods to analyze and compare the proteomes of *T. cruzi* strains may be fundamental to show the association between the parasite's genetic variability and the clinical manifestations resulting from the infection. Profiling the differential proteome of the distinct *T. cruzi* genotypes may shed light on novel approaches for prognosis and clinical management of those affected by Chagas disease.

## Additional file

Additional file 1: **Table S1.** Protein/peptide mass spectrometry data and derived parameters. (XLSX 110 kb)

## Abbreviations

1D: One dimensional
2D: Two-dimensional
ACN: Acetonitrile
ADSS: Adenylosuccinate synthetase
ARG: Arginase
AUC: Area Under Curve
CD: Chagas disease
CHAPS: 3-[(3-cholamidopropyl)dimethylammonio]-1-propanesulfonate
DTT: 1,4-dithiothreitol
DTUs: Chagas disease discrete typing units
DTUs: Discrete typing units
EF1A: Elongation factor 1-alpha
EF2: Elongation factor 2
EIF5A: Eukaryotic translation initiation factor 5a
ETFA: Electron transfer flavoprotein
GAPDH: Glyceraldehyde 3-phosphate dehydrogenase
GOT1: Aspartate aminotransferase
GPX: Glutathione peroxidase-like protein
HAL: Histidine ammonia lyase
HCD: Higher-energy collisional dissociation
HCD2: 3-hydroxyacyl-Coa dehydrogenase
HRF: IgE-dependent histamine-releasing factor
HSP’s: Heat-shock proteins
IPG: Immobilized pH gradient
LIT: Liver infusion triptose
MCP1: Metalocarboxypeptidase1
MDH: Cytosolic malate dehydrogenase
MG: Minas Gerais
MS/MS: Tandem mass spectrometry
MT2598: Peptidase M20/M25/M40
MTHFD1: Tetrahydrofolate synthase
NL: Nonlinear
NO: Nitric oxide
PBS: Sterile buffered saline
PDXK: Pyridoxal kinase
PEPCK: Glycosomal phosphoenolpyruvate carboxykinase
PGFS: Prostaglandin F synthase
PGMI: Phosphoglycerate mutase
PPDK: Pyruvate phosphate dikinase
PSMB’s: Proteasome beta subunits
RAB11: Small GTP-binding protein RAB11
RAB1A: Ras-related protein
RBC: Red blood cell
RGG2: RNA-binding protein RGGm
SDS-PAGE: Sodium dodecyl sulfate polyacrylamide gel electrophoresis
SMP-1: Calpain-like cysteine peptidase
SODB: Iron superoxide dismutase
SPSYN: Spermidine synthase
TAT: Tyrosine aminotransferase
TDR1: Thiol-dependent reductase 1
TFA: Trifluoracetic acid
TP20: Chaperonin TCP20
TUBs: Tubulins
UROC1: Urocanate hydratase
WHO: World Health Organization
